# Reduction of Photoluminescence Quenching by Deuteration of Ytterbium-Doped Amorphous Carbon-Based Photonic Materials

**DOI:** 10.3390/ma7085643

**Published:** 2014-08-06

**Authors:** Hui-Lin Hsu, Keith R. Leong, I-Ju Teng, Michael Halamicek, Jenh-Yih Juang, Sheng-Rui Jian, Li Qian, Nazir P. Kherani

**Affiliations:** 1 Department of Electrical and Computer Engineering, University of Toronto, Toronto, ON M5S 3G4, Canada; E-Mails: huilin.hsu@mail.utoronto.ca (H.-L.H.); keith.leong@mail.utoronto.ca (K.R.L.); michael.halamicek@mail.utoronto.ca (M.H.); 2 Center for Interdisciplinary Science, National Chiao Tung University, Hsinchu 30010, Taiwan; E-Mails: eru7023@gmail.com (I-J.T.); jyjuang@nctu.edu.tw (J.-Y.J.); 3 Department of Materials Science and Engineering, I-Shou University, Kaohsiung 84001, Taiwan; E-Mail: srjian@gmail.com; 4 Department of Materials Science and Engineering, University of Toronto, Toronto, ON M5S 3E4, Canada

**Keywords:** RF-PEMOCVD, fluorinated ytterbium metal-organic compound, deuterated, hydrogenated, amorphous carbon

## Abstract

*In situ* Yb-doped amorphous carbon thin films were grown on Si substrates at low temperatures (<200 °C) by a simple one-step RF-PEMOCVD system as a potential photonic material for direct integration with Si CMOS back end-of-line processing. Room temperature photoluminescence around 1 µm was observed via direct incorporation of optically active Yb^3+^ ions from the selected Yb(fod)_3_ metal-organic compound. The partially fluorinated Yb(fod)_3_ compound assists the suppression of photoluminescence quenching by substitution of C–H with C–F bonds. A four-fold enhancement of Yb photoluminescence was demonstrated via deuteration of the a-C host. The substrate temperature greatly influences the relative deposition rate of the plasma dissociated metal-organic species, and hence the concentration of the various elements. Yb and F incorporation are promoted at lower substrate temperatures, and suppressed at higher substrate temperatures. O concentration is slightly elevated at higher substrate temperatures. Photoluminescence was limited by the concentration of Yb within the film, the concentration of Yb ions in the +3 state, and the relative amount of quenching due to the various de-excitation pathways associated with the vibrational modes of the host a-C network. The observed wide full-width-at-half-maximum photoluminescence signal is a result of the variety of local bonding environments due to the a-C matrix, and the bonding of the Yb^3+^ ions to O and/or F ions as observed in the X-ray photoelectron spectroscopy analyses.

## 1. Introduction

The integration of optical technologies into microelectronic devices has been researched as a viable solution to overcome the speed bottlenecks associated with the ever shrinking of device feature size [1]. Er-implanted in Si, SiO_2_, and ceramic based host thin films [2,3] has been shown to produce photons efficiently from Er^3+^ ions at 1.5 μm, a strategic wavelength for telecommunications. Nevertheless, the ideal optical wavelength for applications on a Si chip and in chip-to-chip communications has not been determined. In order to make use of optical technologies fully, it is essential to explore each alternative Si compatible photonic material. Further, the synthesis techniques of these materials need to be compatible with current Si integrated circuit (IC) fabrication technology. Moreover, it is desirable to develop material growth processes at temperatures below 400 °C in order to meet the Si back end-of-line (BEOL) requirements [4].

Ytterbium (Yb) doped yttrium-aluminum garnet Y_3_Al_5_O_12_ (YAG) single crystals have appeared as promising competitors to the traditional neodymium (Nd) based solid laser for high power diode pumped waveguides [5] and thin-disk lasers [6]. Yb exhibits a simple electronic structure with two manifold levels, a ^2^F_7/2_ ground state and a ^2^F_5/2_ excited state. It has been demonstrated that Yb:YAG lasers lack excited-state absorption, or up-conversion effect owing to a cross-relaxation between the active Yb^3+^ ions [7]. Also, Yb doping concentration can be high due to the enhanced probability of Yb^3+^ substitution of Y^3+^ ions in YAG single crystals [8]. Moreover, Yb has a long luminescence lifetime [9] (up to 1 ms), and a relatively large emission cross section which results in a higher pumping efficiency [10] compared to Nd based solid lasers. However, Yb:YAG single crystals grown via the Czochralski technique possess an inhomogeneous distribution of the active impurities. In particular, the Czochralski technique is not suitable for the fabrication of planar thin film optoelectronic devices integrated on a Si chip platform without convoluted multi-wafer bonding schemes.

Incorporation of Yb into ceramic-based (YAG [11,12,13], Y_2_SiO_5_ (YSO) [14,15,16], KY(WO_4_)_2_ [17], NaLu(WO_4_)_2_ [18], and Al_2_O_3_ [19,20]) wide bandgap semiconductors, oxide-based semiconductors (ZnO [21,22,23], TiO_2_ [24,25], In_2_O_3_ [26]), III–V group based materials (AlN [27]), and Si-based [28] thin film hosts using various deposition techniques, have been shown to produce luminescence at a wavelength of around 1 μm. For Yb doped YAG [11], YSO [14,15], and KY(WO_4_)_2_ [17] thin films, the liquid phase epitaxial (LPE) method has been employed to fabricate homogeneous crystalline films from a molten solute diluted in a solvent. Their growth and post-deposition annealing temperatures are as high as 900–1300 °C so as to avoid growth defects and to further improve the film quality [11,14,15]. In addition, a flat oriented YAG and/or YSO single crystalline substrate is needed as a seed in the LPE method, since growth of the single crystal film is performed by dipping the substrate in a supersaturated melt solution [29]. Consequently, the LPE processing method is not compatible with current Si-based IC fabrication technology.

Lower substrate temperature growth (<300 °C) of amorphous or poly-crystalline/crystalline Yb-doped films (*i.e.*, YAG [13], Y_2_O_3_ [30], Al_2_O_3_ [19,20], CoSb_3_ [31], ZnO [23], ITO [32], AlN [27], and Si-based [28] hosts) and crystalline Yb_2_O_3_ film [33] on Si-based and/or sapphire substrates was achieved via pulsed laser ablation (PLD) [13,19,20,28,30,31,33] and magnetron sputtering [23,27,32] of Yb containing targets. While it is highly desirable to directly employ Si as the host in optoelectronic devices, the bulk crystalline Si bandgap energy is not large enough to activate Yb luminescence. Thus, nanocrystalline silicon (nc-Si) hosts have been applied to act as an efficient sensitizer to transfer photo-induced carriers from the host to the Yb^3+^ ions. Photoluminescence (PL) has been obtained at temperatures ranging from approximately 20–300 K in Yb doped nc-Si films fabricated by ablating a Si:Yb_2_O_3_ mixture target [28]. Moreover, co-doping with O_2_ was highly preferred during the ablation to enhance PL efficiency. Enhancement was achieved through the formation of Yb-O bonds and the reduction in Yb segregation. For PLD grown Yb_2_O_3_ films, room temperature PL was not observed [33]. This was attributed to high Yb concentrations, leading to fast energy migration from one Yb site to another and non-radiative de-excitation. Despite the achievement of lower growth temperatures, the concentration of the Yb and the properties of the host material cannot be independently controlled with pulsed laser ablation and magnetron sputtering techniques. Also, multiple targets with varying Yb composition would be required to obtain different concentrations of Yb-doped films. In addition, a high temperature (>600 °C) post-deposition annealing step is always required to promote the formation of optically active Yb^3+^ ions and to enhance the PL efficiency. This post-deposition annealing step is not amenable with current Si BEOL fabrication technology.

Although extensive research has been performed on Yb-doped ceramic and oxide thin films deposited via various methods, Yb doping in carbon based hosts has been minimal. Yb nanocrystals have been incorporated in various polymers as hybrid materials using sol-gel for potential bio imaging [34,35], gas adsorption, and catalytic activity [36] applications. Hydrogenated amorphous carbon has been used as a host for Er and Yb, however, the attempted PL enhancement of Er via Yb sensitization was not achieved for samples prepared by sputtering of a hybrid Er/Yb/carbon target [37]. An alternative approach for the growth of hydrogenated amorphous carbon (a-C:H) films is by Plasma Enhanced Chemical Vapor Deposition (PECVD) methods, an approach which is compatible with current CMOS fabrication technology [38]. This methodology facilitates integration and allows for reproducible and low-cost films. Additionally, a-C based films possess a number of outstanding properties such as high chemical resistance, mechanical hardness, and transparency in the infrared [38,39]. The specific properties of a-C:H films can be tailored over a wide range by adjusting the amount of *sp*^3^ and *sp*^2^ hybridized carbon and the incorporated hydrogen content in the film via various deposition parameters and methods [39]. These unique and versatile properties provide an impetus to utilize PECVD a-C:H films for specific optoelectronic applications. More importantly, it has been demonstrated that it is possible to prepare carbon based planar waveguides with an attenuation of less than 1 dB/cm [40]. Having low optical losses in photonic materials is one of the most important prerequisites for employing a given material in integrated optics.

In this work, Yb-doped a-C based thin films (a-C(Yb)) were prepared by simple occlusion of the metal-organic compound at low temperatures (<200 °C) in a radio frequency plasma enhanced metal-organic chemical vapor deposition (RF-PEMOCVD) system. The enhancement of PL by substitution of O–H and C–H_x_ bonds with O–D and C–D_x_ bonds is probed and discussed in detail. The effect of the substrate temperature on the PL of a-C(Yb) films is investigated and correlated to the film microstructure and bonding environment. The change in the optical properties of the host a-C upon incorporation of fluorinated Yb metal-organic compound is also discussed.

## 2. Results and Discussion

### 2.1. Photoluminescence Enhancement in a-C(Yb) Film by Deuteration of a-C Host

**Figure 1:**
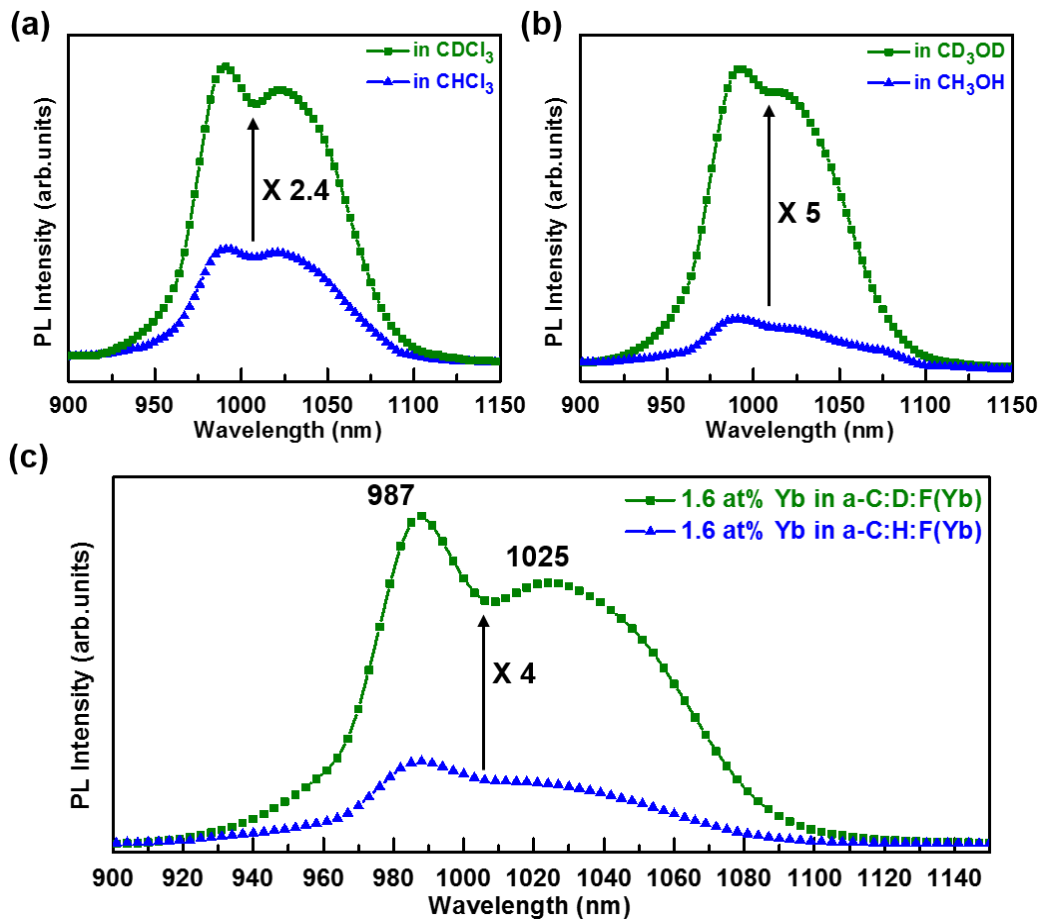
Comparison of the Photoluminescence (PL) intensity of Yb(fod)_3_ powder dissolved in (**a**) chloroform (CHCl_3_) and chloroform-d (CDCl_3_); in (**b**) methanol (CH_3_OH) and methanol-d_4_ (CD_3_OD); (**c**) Comparison of the PL intensity of a-C:D:F(Yb: 1.6 at % ) and a-C:H:F(Yb: 1.6 at %) films prepared using RF power of 60 W, precursor gas flow rate of 20 sccm, deposition pressure of 60 mTorr, substrate temperature of 90 °C, and Yb(fod)_3_ powder evaporation temperature of 110 °C.

**Figure 2:**
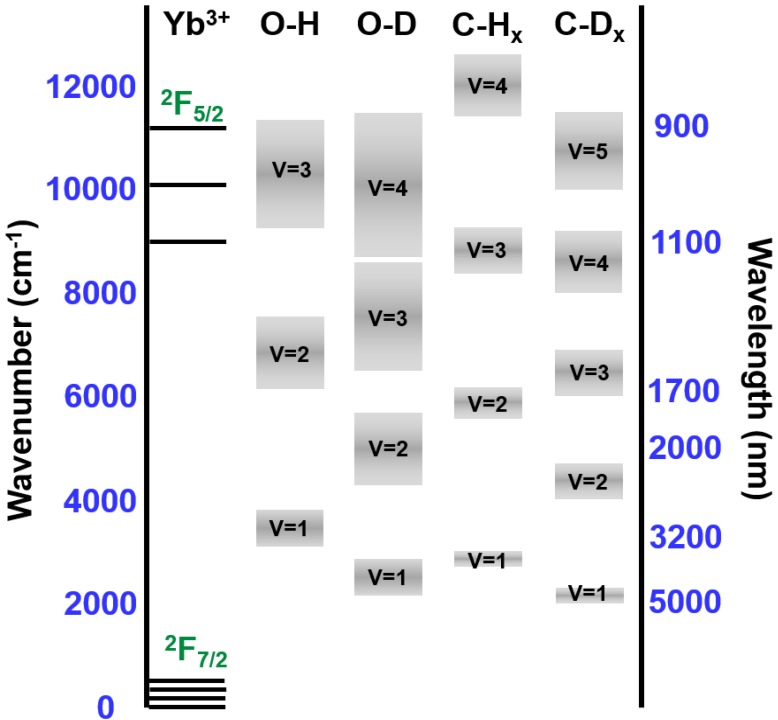
Illustration of energy levels of the vibrational modes for O–H, O–D, C–H_x_, and C–D_x_ bonds in the solid state, where the range of each vibrational mode is inferred from Fourier transform infrared spectroscopy (FTIR) spectra of the host a-C:H, a-C:D and fluorinated a-C:D films (a-C:D:F) as displayed in Figure 3. The grey band here is used to represent the highly varying bonding structure, which reflects the levels associated with the numerous combinations of nearest neighbors and the different local bonding environments.

The bonding environment of the Yb ions in the selected Yb metal-organic compound tris(6,6,7,7,8,8,8-heptafluoro-2,2-dimethyl-3,5-octanedionate) Ytterbium (+III), abbreviated (Yb(fod)_3_), having the chemical structure Yb(C_10_H_10_F_7_O_2_)_3_, is similar to that in Yb_2_O_3_. These optically active Yb^3+^ ions can be preserved under appropriate plasma deposition conditions without the need of a post-deposition annealing step. Fluorine is also incorporated via the partially fluorinated Yb(fod)_3_ precursor. The effect of fluorine on the structure and properties of the films is discussed in Section 2.4. It is noteworthy that the resultant a-C:H:F(Yb) films may contain O–H bonds which form during the decomposition of the Yb(fod)_3_ vapor flux which mixes with the methane (CH_4_) plasma. Although the source of oxygen is limited in the Yb(fod)_3_ precursor, the residual coordinated solvent molecules in the Yb(fod)_3_ compound may also contribute to the overall source of oxygen and hydrogen. In addition, the a-C:H:F(Yb) films will contain C–H bonds where the source of hydrogen includes the methane precursor gas and the existing ligands from Yb(fod)_3_ vapor. Hence, prior to a-C:H:F(Yb) film formation via the RF-PEMOCVD method, the impact of the neighboring C–H_x_ and O–H bonds in the host on the PL of the selected Yb(fod)_3_ precursor was investigated. The 0.5 mM Yb(fod)_3_ powder was dissolved in chloroform (CHCl_3_), methanol (CH_3_OH), and their respective deuterated solvents, *i.e.*, chloroform-d (CDCl_3_) and methanol-d_4_ (CD_3_OD). It can be seen in Figure 1a that the PL intensity is enhanced by a factor of 2.4 as the solvent CHCl_3_ is replaced with CDCl_3_. This suggests the Yb(fod)_3_ PL is quenched by the C–H bonds in neighboring solvent molecules. Figure 1b displays the PL enhancement by a factor of five when using the CD_3_OD solvent instead of the CH_3_OH solvent. Since the CH_3_OH host solvent molecules contain both C–H_x_ and O–H bonds, the PL quenching of Yb(fod)_3_ is more significant. As previously shown, O–H bonds contribute non-radiative deactivation channels for excited Yb^3+^ ions [41,42,43]. It has been demonstrated that the Yb radiative lifetime is limited by the presence of O–H groups in the sol-gel silica glass host matrix [42]. Hence, hydrogen containing groups such as X–H (where X = O, C) appear to aid in PL quenching due to the proximity of their vibrational modes to the excited states of Yb ions as indicated in Figure 2. To suppress the O–H and C–H_x_ quenching effect near the Yb^3+^ ions in a-C:H:F(Yb) films, the precursor gas was changed from methane (CH_4_) to deuterated methane (CD_4_). This reduces the formation of O–H and C–H_x_ bonds which are now substituted by O–D and C–D_x_ bonds [44,45].

The room temperature PL spectra of a-C(Yb) samples exhibit peaks around 987 and 1025 nm, shown in Figure 1c, which corresponds to the ^2^*F*_5/2_ to ^2^*F*_7/2_ electronic transition of Yb^3+^ ions in Figure 2. The spectral width of the emission band is attributed to inhomogeneous and homogeneous broadening in addition to Stark splitting of the Yb^3+^ excited and ground states. Its full width at half-maximum (FWHM) is approximately 85 nm, suggesting the potential of enabling a wide gain bandwidth for optical amplification and tunable laser design. However, the FWHM of ~85 nm in the a-C(Yb) samples is slightly smaller than that obtained from the Yb(fod)_3_ containing solutions shown in Figure 1a,b (~90 nm). This suggests the Yb^3+^ state is relatively preserved compared to that of the pristine Yb(fod)_3_ compound in solution, a highly disordered local environment. In addition, the PL peak of the a-C(Yb) samples is wider than those of other Yb doped ceramic and oxide based hosts [13,25,46,47]. This suggests the Yb^3+^ ion possesses a variety of local bonding environments in the a-C matrix, which will be discussed further in the X-ray photoelectron spectroscopy (XPS) analyses in Section 2.4. Figure 1c shows the PL intensity is enhanced by a factor of four for the same Yb concentration in the deuterated a-C matrix as opposed to the hydrogenated a-C matrix. This confirms that Yb photoluminescence is enhanced through the substitution of O–H and C–H_x_ groups with O–D and C–D_x_ groups in the a-C(Yb) films prepared by the RF-PEMOCVD method. Nevertheless, the enhanced luminescence factor for the deuterated a-C(Yb) sample is lower than that of the Yb(fod)_3_ deuterated methanol solution as shown in Figure 1b. The factors that may contribute to the lower PL enhancement for the deuterated a-C(Yb) sample include the presence of C–H_x_ bonds within the host that may reside in close proximity to the Yb^3+^ ions for the given deposition conditions and/or some of the Yb ions in a-C(Yb) not being in the optically active 3+ state which is discussed in Section 2.2 and presented in Table 1.

**Table 1:** Ratios of atomic concentrations and relative/absolute atomic concentrations of relevant elements in as-received stoichiometric Yb(fod)_3_ compound, and in six a-C:D:F(Yb) films deposited under varying conditions as determined from XPS measurements.

Sample	Substrate temperature (°C)	C at %	O at %	F at %	Yb at %	[O]/[Yb]	[F]/[Yb]	[C]/[Yb]	[O]/[C]	[F]/[C]	[O]/[F]	Thickness (100 nm)
Yb(fod)_3_		34.1	6.8	23.9	1.1	6.00	21.00	30.00	0.20	0.70	0.29	
1	60	48.5	2.7	45.2	3.6	0.75	12.72	13.64	0.06	0.93	0.06	33.21
2	70	59.9	2.5	35.0	2.6	0.94	13.23	22.64	0.04	0.58	0.07	27.65
3	80	63.4	3.6	30.9	2.1	1.68	14.61	29.94	0.06	0.49	0.12	20.23
4	90	68.9	3.2	26.3	1.6	1.99	16.25	42.63	0.05	0.38	0.12	14.52
5	120	86.4	7.2	5.5	0.9	8.00	6.05	95.73	0.08	0.06	1.32	5.67
6	150	91.8	6.1	1.7	0.4	17.50	4.84	261.64	0.07	0.02	3.62	3.51

**Figure 3:**
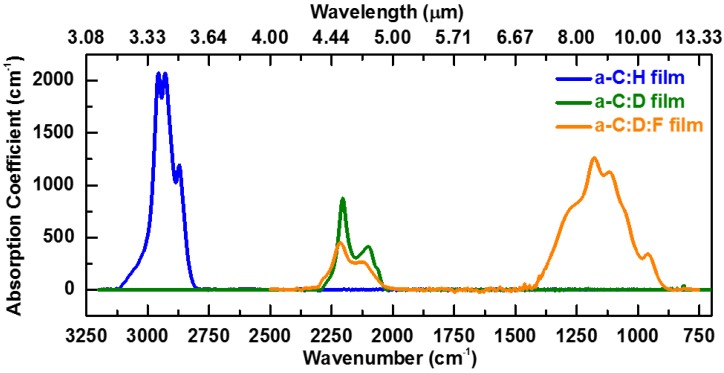
FTIR spectra of the host a-C:H, a-C:D, and a-C:D:F (CF_4_/[CF_4_ + CD_4_] = 50 vol.%) films without Yb metal-organic compound incorporation prepared under the same deposition condition except for the precursor gas. The relative wavenumber/wavelength positions and the range of the first vibrational mode illustrated in Figure 2 are inferred from this data.

The enhancement of PL is primarily attributed to the weaker interaction strength between Yb^3+^ and the O–D fourth harmonic vibration (υ = 4) and the C–D_x_ fourth and fifth harmonic vibrations (υ = 4, 5) as compared to the interaction strength between Yb^3+^ and the O–H third harmonic vibration (υ = 3) and the C–H_x_ third and fourth harmonic vibrations (υ = 3, 4), as referred in Figure 2. The O–H third harmonic vibrational mode (at approximately 9300–11,400 cm^−1^ [41,48]) matches the radiative transition from the first excited state ^2^*F*_5/2_ to the ground state ^2^*F*_7/2_ in Yb^3+^ ions (at approximately 9090–11,110 cm^−1^ as determined from the PL spectrum in Figure 1c), as shown in Figure 2. Also, the C–H_x_ third and fourth harmonic vibrational modes (at approximately 8400–9300 cm^-1^ and 11,200–12,400 cm^−1^ as inferred from FTIR spectra of C–H_x_ peaks measured in a-C:H film as displayed in Figure 3) match the radiative transitions in Yb^3+^ ions. The band indicated in Figure 2 is used to represent the highly varying bonding structure in the a-C matrix, which reflects the levels associated with the numerous combinations of nearest neighbors and the different local bonding environments. Accordingly, if excited Yb^3+^ ions are disturbed by O–H and C–H_x_ oscillators, a non-radiative transition occurs. Considering the undistorted oscillator model [49], the transition probability between Yb^3+^ and the O–D and C–D_x_ vibrational modes of the host deuterated a-C film is lower than that between Yb^3+^ and the O–H and C–H_x_ vibrational modes. This leads to an increase in the efficiency of the PL for a-C:D:F(Yb) films as shown in Figure 1c. The 8th and 9th harmonic vibrational modes of C–F_x_ overlap with the Yb^3+^ first excited state, however they play a negligible role on PL quenching as these vibrational modes are much higher (υ = 8, 9) compared to O–H (υ = 3) and C–H_x_ (υ = 3, 4) modes. Hence, by selecting the Yb(fod)_3_ metal-organic compound as the doping precursor instead of Yb(tmhd)_3_, tris(2,2,6,6-tetramethyl-3,5-heptanedionato)Ytterbium(+III) with chemical structure Yb(C_11_H_19_O_2_)_3_, the PL quenching by C–H_x_ is expected to be mitigated.

### 2.2. Effect of Substrate Temperature on a-C:D:F(Yb) Film

The temperature of the growth surface was found to influence the concentration of Yb occluded in the a-C:D:F(Yb) films, and hence the PL efficiency. Table 1 lists the ratios of the atomic concentrations and the relative (and absolute) atomic concentrations of the relevant elements in the as-received stoichiometric Yb(fod)_3_ compound, and in the six a-C:D:F(Yb) films deposited under varying substrate temperatures as determined from XPS measurements. In comparing the a-C:D:F(Yb) film deposited at a substrate temperature of 60 °C to the stoichiometric Yb(fod)_3_ compound, a few results are observed. The [O]/[Yb], [F]/[Yb], and [C]/[Yb] ratios are approximately 8×, 1.65×, and 2.2× smaller, respectively, in the film. This suggests that Yb incorporation is being promoted in the film. Moreover, fluorine incorporation is being promoted since the [O]/[F] ratio is 4.8× larger in the film. The increase in the F concentration is attributed to the selective incorporation of C*_m_*F*_n_* fragments. This film also exhibits a surprising reduction in the amount of bonded carbon as witnessed by the lower [C]/[Yb] and the higher [F]/[C] ratios. One would expect the carbon concentration to increase since carbon can be sourced from CD_4_ or the Yb(fod)_3_ precursor. Evidently, significant dissociation of the Yb(fod)_3_ compound occurs in the plasma environment. This is supported by the large variation in the atomic ratios ([C]/[Yb] = 13.64, [O]/[C] = 0.06, [F]/[C] = 0.93), as compared to the stoichiometric samples ([C]/[Yb] = 30, [O]/[C] = 0.20, [F]/[C] = 0.7). The bond energies may provide a possible explanation for the relative exclusion of C and O in the a-C:D:F(Yb) film grown at the substrate temperature of 60 °C. The C–C, C–O, and Yb–O bonds are expected to have a lower bond energy than C-F, C=C, and C=O bonds. Hence, it is expected that they will be heavily dissociated within the plasma and as a result may not be incorporated in the resultant film.

**Figure 4:**
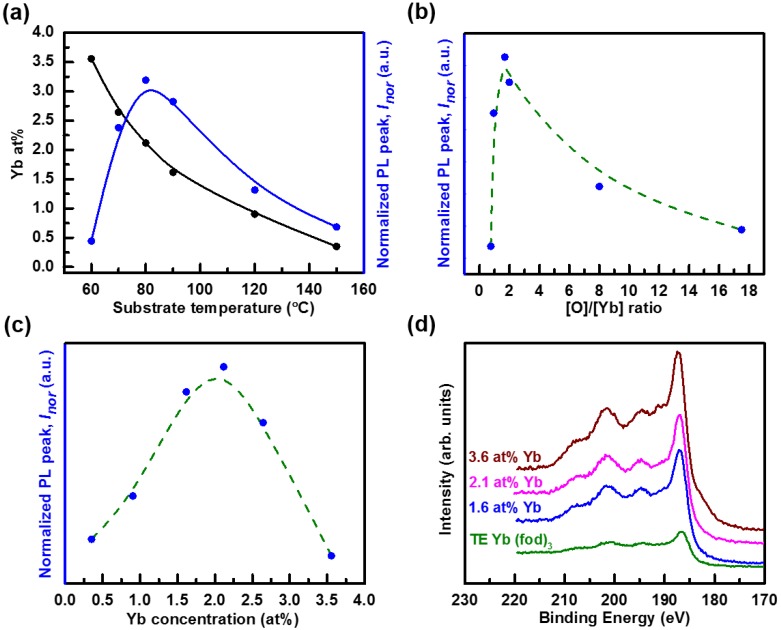
(**a**) The Yb concentration (solid black circle) and normalized PL peak intensity, *I_nor_*, (solid blue circle) as a function of the substrate temperature for a-C:D:F(Yb) films deposited at an RF power of 60 W. The normalized PL peak intensity is shown to depend critically on the (**b**) [O]/[Yb] ratio; and (**c**) [Yb]. The lines are guides to the eye. *I*_nor_ is defined as the PL intensity peaking at ~987 nm normalized to the a-C:D:F(Yb) film thickness; (**d**) Yb4d XPS spectra of the three a-C:D:F(Yb) films, and TE Yb(fod)_3_ film (thermally evaporated Yb(fod)_3_ compound in the vacuum chamber with CD_4_ precursor gas flowing without plasma ignition). The curves have been shifted vertically for clarity of presentation.

Analyzing the XPS spectra for the films grown as the substrate temperature was varied from 60 to 150 °C yields a number of results. The [F] and [Yb] decrease monotonically with increasing substrate temperature. The [C] increases monotonically while the [O] shows a slightly increasing trend with increasing substrate temperature. The considerable loss of [F] and [Yb] with increasing substrate temperature is associated with a drop in the deposition of C_m_F*_n_*, YbO*_x_*C*_m_*F*_n_*, Yb, and/or YbO*_x_* molecular fragments. The relative variation in the drop of the [F] and [Yb] may be due to the temperature dependence of the sticking coefficient or the desorption rate of Yb-containing fragments and F-containing fragments. Despite the decreasing [Yb] with increasing substrate temperature (black curve in Figure 4a), the relative PL intensity increases, peaks at a substrate temperature of 80 °C, and then decreases as indicated in blue curve of Figure 4a. The increasing PL intensity with substrate temperature is attributed to the increasing O concentration and hence an increasing [O]/[Yb] ratio as seen in Figure 4b. Hence, more Yb atoms are considered to be in the +3 state. Above a substrate temperature of 80 °C the PL intensity decreases even though the [O]/[Yb] ratio continues to increase. This is due to the decreasing [Yb] with increasing substrate temperature (Figure 4a). At a substrate temperature of 150 °C, the majority of the film is a-C:D with minute F and Yb concentrations as indicated in Table 1. A summary of the variation of the PL intensity with Yb concentration in a-C:D:F matrix is shown in Figure 4c. *I*_nor_ increases linearly up to Yb concentration of approximately 2.1 at % and thereafter begins to drop until 3.6 at % at which point the PL becomes very weak. The latter suggests a reduction in lifetime Γ beyond this point [50] as result of energy transfer between optically active Yb^3+^ and Yb^3+^ ion pairs.

### 2.3. Yb Oxidation State in a-C:D:F(Yb) Film

The Yb4d XPS spectra of the thermally evaporated (abbreviated TE) Yb(fod)_3_ film, and three a-C:D:F(Yb) samples with different Yb concentrations are compared in Figure 4d. Yb ions appear in the +2 state in the metal and in the +3 state or slightly mixed +2 and +3 states in the sesquioxide. Yb^2+^ metal clusters show two distinct peaks located at 183 and 192 eV [51,52]. Also, the partially oxidized metal shows the two distinct peaks, as well as a small peak around 186–187 eV [52]. However, the fully oxidized Yb in the +3 state shows only one main peak around 186–187 eV with the broad peaks in the higher-binding energy region [51,52]. The broad peaks are attributed to the 4d levels in the Yb^3+^ ions forming multiplet splitting with electro-static coupling of a 4d photohole to the unfilled valence shell of Yb^3+^(4*f*^13^) [51,53]. From Figure 4d, the spectra reveal similar characteristics for all of the samples, implying the local environment of the incorporated Yb in a-C:D:F(Yb) films is similar to the Yb in the pristine Yb(fod)_3_ compound. More importantly, it confirms the preservation of the Yb^3+^ state in the a-C(Yb) samples. However, the fact that the [O]/[Yb] ratio is less than six (shown in Table 1), as is the case for the pristine Yb(fod)_3_ powder, suggests that some Yb ions may have undergone a shift in their electronic structure (the ^2^*F*_5/2_ and/or ^2^*F*_7/2_ energy levels) or may be optically inactive. A shift in the electronic structure would be supported by the larger width of the observed PL signal as compared to the reported widths from Yb doped ceramic [11,14,18,30], oxide [21,22,24,25] based host films, and Yb_2_O_3_ crystalline films [33]. This wider luminescence spectra were also observed in Yb doped AlN films, where the host AlN films are essentially amorphous in form with AlN nanocrystals embedded in the matrix [54]. Nevertheless, the Yb4d XPS spectra suggest the Yb^3+^ state is mostly preserved in the a-C:D:F(Yb) films, leading to the observed PL.

### 2.4. Structural Analyses of a-C:D:F(Yb) Film

**Figure 5:**
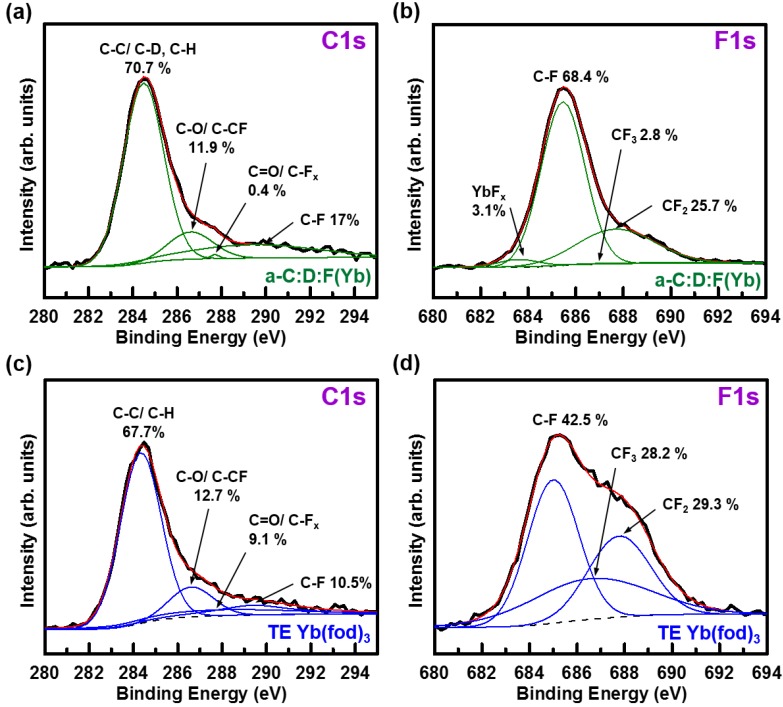
Deconvoluted XPS (**a**) C1s and (**b**) F1s spectra of the a-C:D:F(Yb) film with maximum *I*_nor_ prepared using RF power of 60 W, precursor gas flow rate of 20 sccm, deposition pressure of 60 mTorr, substrate temperature of 80 °C, and Yb(fod)_3_ powder evaporated at a temperature of 110 °C; and (**c**) C1s and (**d**) F1s spectra of the TE Yb(fod)_3_ film.

Detailed XPS analyses were performed to examine the bonding details of the resultant a-C:D:F(Yb) film. In Figure 5a, the deconvoluted C1s spectra indicate the majority of carbon bonds are C–C and/or C–D/C–H, with some C–F, C–O and/or C–CF, and very few C=O and/or C–F_x_ bonds in the a-C:D:F(Yb) film. The deconvoluted F1s spectra displayed in Figure 5b show a small concentration of YbF_x_ bonds exist in the a-C:D:F(Yb) film, while the majority of F atoms are bonded to carbon as C–F bonds, with some C–F_2_ bonds and a minute amount of C–F_3_ bonds. Note that the fitting error (Ʃ χ^2^) would be 33% higher without adding the YbF_x_ bond peak at 683.6 eV during the F1s deconvolution process. This suggests that the probability of Yb bonding in the optically active +3 state could be increased by formation of the Yb–F_x_ bonds, in addition to the expected +3 state as in the pristine Yb–O bonds of the Yb(fod)_3_ compound. This additional bonding environment could further promote the luminescence efficiency of the a-C(Yb) film, as demonstrated by the observed luminescence from YbF_3_ nanoparticles [55,56,57]. The different percentages of carbon and fluorine related bonds, as depicted in Figure 5, imply that significant dissociation of the Yb(fod)_3_ compound occurs in the plasma environment. For instance, plasma dissociation of the C–F_3_ and C–F_2_ groups into C–F is shown by the decrease of the F1s C–F_3_ and C–F_2_ peaks in the a-C:D:F(Yb) film in Figure 5b compared to the TE Yb(fod)_3_ film in Figure 5d. This is also supported by the increase in the C–F bond for the C1s peak, as well as the decrease in the C=O and/or C–F_x_ and C–O and/or C–CF peak, relative to the TE Yb(fod)_3_ film. Note that no C1s C–Yb peak was observed. Thus, the resultant a-C:D:F(Yb) film contains Yb^3+^ ions bonded to O and/or F (*i.e.*, YbF_3_, YbFO_2_, YbF_2_O, and YbO_3_), and is surrounded by a variety of local environments (with varying sizes of the Yb^3+^ sites) due to the structural flexibility of the a-C matrix. Consequently, these structural observations may confirm the observed wider FWHM PL signal shown in Figure 1c, compared to other Yb doped ceramic [11,14,18,30] and oxide based materials [21,22,24,25].

The XPS measurements revealed that some samples contained a high fraction of fluorine. In order to probe the changes in the optical properties of the host a-C film due to fluorination, fluorinated a-C:D (a-C:D:F) host films were prepared from deuterated methane (CD_4_) and tetrafluoromethane (CF_4_) mixtures. It was observed that as the relative F at % increases (*i.e.*, CF_4_/[CF_4_ + CD_4_] ratio increases), the *E*_04_ optical bandgap of the resultant a-C:D:F films decreases (data now shown here). Robertson [39] describes the microstructures of a-C as a continuous network of *sp*^3^ bonded carbon atoms (predominately C–C or a mixture of C–C and C–H) with *sp*^2^ bonded carbon localized clusters (the number and size of the C=C) embedded within the network. The *sp^3^* bond configuration forms σ–σ* bands and the *sp*^2^ sites create π–π* bands by forming the localized states. The size and quantity of the *sp*^2^ clusters dominate the film’s optical properties. Accordingly, the increasing %*sp*^2^ content in the film implies an increase in the localized density of states lying deeper in the gap. Thus, the decrease in the optical bandgap *E*_04_ as the relative F at % increases in the films could be attributed to the increase in %*sp*^2^ bonding in the film [58,59,60].

Although the *E*_04_ of the a-C(Yb) films cannot be accessed at this moment, samples 1 and 2, as shown in Table 1, represent a-C(Yb) films grown at the substrate temperature of 60 and 70 °C, and along with the PL shown in Figure 4a, it could be inferred that it appears that these films have lower *E*_04_ compared to that of sample 3. This is due to higher incorporation of F [58,59,60] and Yb [61,62] concentrations than those in sample 3 as seen in Table 1. As the relative F at % increases in the films the *E*_04_ optical bandgap was observed to decrease (data not shown here), which could be attributed to the increase in %*sp*^2^ bonding in the film giving rise to a localized density of states lying deeper in the gap [58,59,60]. Moreover, with higher Yb incorporation, %*sp*^2^ is expected to further increase considering the observations of Er doped or Er oxide doped diamond like carbon films reported by Foong *et al.* [61,62]. Thus the higher %*sp*^2^ in samples 1 and 2 of the a-C(Yb) films might be another minor factor that contributes toward the lower PL intensity observed in addition to the concentration quenching effect from the high Yb concentration. Accordingly, higher %*sp*^2^ could result in a higher probability of photoelectrons for non-radiative recombination in the a-C:D host, leading to a lower probability of photoelectrons transferring from the a-C:D host to Yb^3+^ ions and further emitting 1 µm photons at room temperature. With respect to the change in film optical properties after incorporation of the Yb compound, the refractive index *n* and extinction coefficient *k* are expected to increase based on the results obtained from rare-earth ions doped PbTiO_3_ thin films prepared by sol-gel method [63]. The role of %*sp*^2^ (the number and size of the C=C) and energy transfer mechanism between Yb^3+^ ions and the host a-C are still under investigation via application of multiple laser excitations (488 and 980 nm) in addition to the presently employed 532 nm laser source.

## 3. Experimental Section

### 3.1. RF-PEMOCVD and Sample Preparation

A capacitively coupled RF-PEMOCVD system was deployed to deposit a-C(Yb) films. An ac-powered thermal evaporator situated next to the RF-powered electrode, inside the deposition chamber was utilized to *in situ* dope the Yb metal-organic compound while the a-C film deposition proceeds. The technique has been demonstrated to provide the capability of doping Er in a vertically uniform profile. As well, this technique provides independent control of the Er concentration profile and the optical properties of the host material [44,64].

For a-C(Yb) films, the substrate temperature was varied from 60 to 150 °C. The hydrocarbon gas flow rate was 20 sccm, chamber pressure was at 60 mTorr, and the RF power was kept constant at 60 W. Double-side polished crystalline silicon substrates with resistivity of more than 1000 ohm-cm were subjected to the standard CMOS cleaning procedure before being loaded into the chamber. Methane (CH_4_) with purity of 99.999% and tetrafluoromethane (CF_4_) with purity of 99.996% were purchased from Linde Canada Limited. Deuterated methane (CD_4_) with isotopic purity of 99 atom % D was purchased from Sigma-Aldrich (St. Louis, MO, USA).

### 3.2. Incorporated Yb Metal-Organic Compound

**Figure 6:**
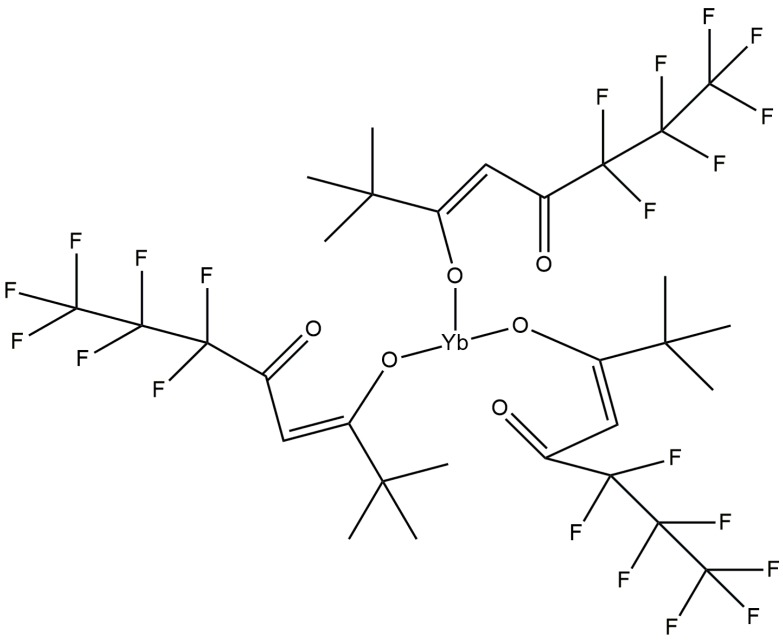
Illustration of the Yb metal-organic compound, Yb(fod)_3_, with chemical structure Yb(C_10_H_10_O_2_F_7_)_3_.

The Yb metal-organic compound tris(6,6,7,7,8,8,8-heptafluoro-2,2-dimethyl-3,5-octanedionate) Ytterbium (+III), abbreviated (Yb(fod)_3_), having the chemical structure Yb(C_10_H_10_F_7_O_2_)_3_ as illustrated in Figure 6, was selected as the doping candidate for a-C(Yb) films. The Yb in the Yb(fod)_3_ compound is coordinated by six oxygen atoms, which represents a similar bonding environment to Yb(tmhd)_3_. Yb(tmhd)_3_ has been employed as the precursor to dope Yb_2_O_3_ in Y_2_O_3_ films by atomic layer deposition method [65] in order to maintain the optically active Yb^3+^ state. Accordingly, it was presumed that the optically active Yb^3+^ ions can be preserved under appropriate deposition conditions in the present study. In addition, it has been previously demonstrated that the Er^3+^ optical activity can be partially preserved without the need of a post-deposition annealing step by utilizing the Er(fod)_3_ compound in the RF-PEMOCVD method [44,64]. The hydrocarbon ligands of the Yb metal-organic compound are expected to promote a high Yb concentration in the a-C host matrix as its structure matches the internal structure of the a-C host matrix. In this work, a controlled vapor flux of the Yb(fod)_3_ compound is introduced by thermal evaporation at 110 °C. The vapor flux is mixed with the hydrocarbon plasma for a-C(Yb) film formation. The Yb(fod)_3_ powder was obtained from Strem Chemicals Inc. (Newburyport, MA, USA) and was loaded into the vacuum chamber without any special treatment.

### 3.3. Film Characterization

#### 3.3.1. Photoluminescence

Photoluminescence spectra of a-C(Yb) films were collected at room temperature to verify the luminescence property of the Yb in the a-C(Yb) films. A continuous wave diode-pumped solid-state 532 nm laser with a power density of 20 mW/mm^2^ was used as the excitation source. A thermoelectrically cooled InGaAs photodiode, 800–1700 nm detection range, with standard lock-in technique was employed. A single pass monochromator with resolution bandwidth of 2 nm was utilized to disperse the emitted PL. *I*_nor_ indicated in Figure 4 is defined as the PL intensity peaking at ~987 nm and normalized to the respective a-C(Yb) film thickness indicated in Table 1.

#### 3.3.2. X-ray Photoelectron Spectroscopy

X-ray photoelectron spectroscopy (XPS) was employed to quantitatively characterize the elemental composition and chemical structure of a-C(Yb) films. The XPS spectra were collected from the surface of the sample using a monochromatic Al Kα X-ray source in a Thermo Scientific K-Alpha spectrometer (ThermoFisher Scientific, Waltham, MA, USA) with an ultrahigh vacuum of the order of 10^−9^ Torr. The spectra were calibrated relative to a charge reference of adventitious carbon with a core level binding energy of 284.5 eV, while for curve fitting we used published values of binding energies and Gaussian decompositions with Smart background.

#### 3.3.3. Fourier Transform Infrared Spectroscopy

The relative wavenumber/wavelength positions and range of energy levels of the first vibrational modes (υ = 1) of C–H_x_, C–D_x_, and C–F_x_ bonds, shown in Figure 2, were obtained from the absorption peaks of the host a-C:H, a-C:D, and a-C:D:F films measured by Fourier Transform Infrared Spectroscopy (FTIR) technique as displayed in Figure 3. From Figure 3, it can be seen that the first vibrational modes (υ = 1) of C–H_x_ and C–D_x_ bonds are located at 2800–3100 cm^−1^ (blue curve) and 2000–2300 cm^−1^ (green curve). Thus, it can be inferred that the third (υ = 3) and fourth (υ = 4) harmonic vibrational modes of C–H_x_ peaks are at approximately 8400–9300 cm^−1^ and 11,200–12,400 cm^−1^, which match the radiative transition from the first excited state ^2^*F*_5/2_ to the ground state ^2^*F*_7/2_ in Yb^3+^ ions (at approximately 9090–11,110 cm^−1^ as determined from the PL spectrum in Figure 1c) as shown in Figure 2. Also, the fourth and fifth harmonic vibrations (υ = 4, 5) of C–D_x_ bonds can be inferred to be at 8000–9200 cm^−1^ and 10,000–11,500 cm^−1^, which match the radiative transition in Yb^3+^ ions as well. The first vibrational modes (υ = 1) of O–H and O–D are approximately located at 3100–3800 cm^−1^ centered at 3450 cm^−1^ and 2150–2850 cm^−1^ centered at 2500 cm^−1^ from the cited reference papers [41,48]. Thus, it can be inferred that the O–H third harmonic vibrational mode (υ = 3) is at approximately 9300–11,400 cm^−1^ and the O–D third and fourth harmonic vibrational mode (υ = 3, 4) are at approximately 6450–8550 cm^−1^ and 8600–11,400 cm^–1^.

Furthermore, the first set of harmonic vibrational mode of C–F_x_ is at 900–1400 cm^−1^ as observed in Figure 3 (orange curve). Thus, it can be inferred that the 8th and 9th harmonic vibrational modes are at 7200–11,200 cm^−1^ and 8100–12,600 cm^−1^ (not shown in Figure 2), which also overlap with the Yb^3+^ first excited state. However, they play a negligible role on PL quenching as these vibrational modes are much higher (υ = 8, 9) compared to O–H (υ = 3) and C–H_x_ (υ = 3, 4) modes. Moreover, the darker sections of the band in Figure 2 signify a higher absorption coefficient. Hence, the dark band indicates the energy distribution of the vibrational levels.

The FTIR spectra were characterized using a Perkin Elmer 2000 spectrometer (PerkinElmer, Waltham, MA, USA) with a resolution of 4 cm^−1^. The transmission spectra were background corrected for the interference patterns emerging due to multiple reflections in the film. The absorption was determined using the following relation:

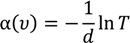
(1)
where α(υ) is the absorption coefficient; *d* is the thickness of the film; and *T* is the normalized transmission of the a-C film with the background removed.

#### 3.3.4. Spectroscopic Ellipsometry

The optical bandgaps *E*_04_ of the host deuterated/fluorinated amorphous carbon (a-C:D:F) films were probed through spectroscopic ellipsometry. The measurements were carried out using an UV-Vis-NIR spectroscopic ellipsometer (Semilab, Budapest, Hungary). The spectra were analyzed by regression fitting using the linear Levenberg-Marquardt algorithm method with a maximum of 1000 iterations using a three-layer optical structure comprising void (ambient)/a-C layer/c-Si substrate. The five constants of Forouhi-Bloomer dispersion model [66] and thickness of the a-C layer were allowed to vary during the fitting process. The optical bandgap *E*_04_, defined as the photon energy at which the absorption coefficient α(=4π*k*/λ) is equal to 10^4^ cm^−1^, was determined from the extinction coefficient *k*; λ is the wavelength.

## 4. Conclusions

The *in situ* growth of Yb-doped a-C thin films on Si substrates at low temperature (<200 °C) was successfully demonstrated by a simple one-step RF-PEMOCVD system. Room temperature PL at a wavelength of around 1 µm was observed from these a-C(Yb) films. A subsequent high temperature post-deposition annealing procedure was not required. This is a result of the direct incorporation of the optically active Yb^3+^ ions from the specially selected Yb(fod)_3_ metal-organic compound. The PL was enhanced by a factor of four due to the use of deuteration as opposed to hydrogenation of the a-C host. This served to lower the interaction strength between the excited Yb^3+^ and the neighboring harmonic vibrational modes. Moreover, fluorination of the a-C host may assist in the suppression of the PL quenching effect through the partial substitution of C–H bonds with C–F bonds.

The substrate temperature was varied from 60 to 150 °C, and was shown to greatly influence the relative deposition rates of the plasma dissociated metal-organic species. It was observed that the relative PL intensity increases, peaks at a substrate temperature of 80 °C, and then decreases. The increasing PL intensity is attributed to an increase in [O]/[Yb] ratio, leading to more Yb atoms in the +3 state. Above a substrate temperature of 80 °C the [O]/[Yb] ratio continues to increase, however the PL decreases. This is due to the considerable loss of the [Yb], which is associated with a drop in the temperature-dependent deposition of YbO*_x_*C*_m_*F*_n_*, Yb, and/or YbO*_x_* molecular fragments. The relative PL intensity increases linearly with the Yb concentration up to a [Yb] of approximately 2.1 at cm^−1^. Thereafter the PL intensity begins to drop, suggesting a severe energy transfer between active Yb^3+^ and Yb^3+^ ion pairs beyond this point.

The Yb4d XPS spectra indicated that the environment of the incorporated Yb in a-C(Yb) films is similar to the Yb in the pristine Yb(fod)_3_ compound. Although, the fact that the [O]/[Yb] ratio is less than six suggests some Yb ions may have their electronic structure shifted (the ^2^*F*_5/2_ and/or ^2^*F*_7/2_ energy levels) or have become optically inactive. By examining the C1s and F1s deconvoluted XPS spectra in detail, the Yb^3+^ ions in the a-C(Yb) films are found to be bonded to O and/or F (*i.e.*, YbF_3_, YbFO_2_, YbF_2_O, YbO_3_). The formation of the Yb-F_x_ bonds increases the probability of Yb bonding in the optically active +3 state, which may promote the luminescence efficiency of a-C(Yb) films. However, the relatively high F concentration in the host a-C matrix could lead to an increase in the de-excitation pathways. This is due to the smaller band gap or the increased states within the band gap typically found in a-C:F materials.

In summary, the a-C:D:F(Yb) films synthesized by the RF-PEMOCVD method at low temperatures (<200 °C), exhibit room temperature luminescence owing to direct incorporation of Yb^3+^ ions. Also, luminescence is enhanced by the substitution of C–D_x_, C–F_x_, and O–D bonds with C–H_x_ and O–H bonds. The wide FWHM observed is due to the various local bonding environments. These properties suggest that a-C:D:F(Yb) may be a promising material for active photonic devices to be integrated with the current Si CMOS platform.
